# Leveraging PEPFAR-Supported Health Information Systems for COVID-19 Pandemic Response

**DOI:** 10.3201/eid2813.220751

**Published:** 2022-12

**Authors:** Muzna Mirza, Yoran Grant-Greene, Marie P.J.S. Valles, Patrice Joseph, Stanley Juin, Stephan Brice, Patrick Dely, Marie G.R. Clement, Manish Kumar, Meredith Silver, Samuel Wambugu, Christopher Seebregts, Daniel Futerman, Fitti Weissglas, Veronica Muthee, Wendy Blumenthal, Tadesse Wuhib, Steven Yoon, Daniel H. Rosen

**Affiliations:** US Centers for Disease Control and Prevention (CDC), Atlanta, Georgia, USA (M. Mirza, W. Blumenthal, T. Wuhib, S. Yoon, D.H. Rosen);; CDC Country Office, Port-au-Prince, Haiti (Y. Grant-Greene, M.P.J.S. Valles, P. Joseph, S. Juin, S. Brice);; Ministry of Public Health and Population, Port-au-Prince (P. Dely, M.G.R. Clement);; PATH, University of North Carolina, Chapel Hill, North Carolina, USA (M. Kumar, M. Silver);; PATH Consortium, Seattle, Washington, USA (S. Wambugu);; Jembi, Cape Town, South Africa (C. Seebregts, D. Futerman);; University of California San Francisco, San Francisco, California, USA (F. Weissglas, V. Muthee)

**Keywords:** COVID-19, respiratory infections, severe acute respiratory syndrome coronavirus 2, SARS-CoV-2, SARS, coronavirus disease, zoonoses, viruses, coronavirus, health information systems, public health surveillance, health information exchange, electronic medical record, EMR, PEPFAR, Haiti

## Abstract

Since 2003, the US President’s Emergency Plan for AIDS Relief (PEPFAR) has supported implementation and maintenance of health information systems for HIV/AIDS and related diseases, such as tuberculosis, in numerous countries. As the COVID-19 pandemic emerged, several countries conducted rapid assessments and enhanced existing PEPFAR-funded HIV and national health information systems to support COVID-19 surveillance data collection, analysis, visualization, and reporting needs. We describe efforts at the US Centers for Disease Control and Prevention (CDC) headquarters in Atlanta, Georgia, USA, and CDC country offices that enhanced existing health information systems in support COVID-19 pandemic response. We describe CDC activities in Haiti as an illustration of efforts in PEPFAR countries. We also describe how investments used to establish and maintain standards-based health information systems in resource-constrained settings can have positive effects on health systems beyond their original scope.

Since its creation in 2003, the US President’s Emergency Plan for AIDS Relief (PEPFAR) has funded and supported development, implementation, and expansion of capabilities, and maintenance of health infrastructure, including health information systems, for HIV/AIDS and related diseases, such as tuberculosis (TB), in numerous countries (*1*). When the COVID-19 pandemic emerged, several PEPFAR countries were already extensively using health information systems for managing, reporting, and visualizing the burden of HIV/AIDS and TB among their populations.

As with any public health emergency response, the COVID-19 pandemic response required accurate, standards-based, and timely public health data for optimal national prevention, detection, and response efforts (*2*–*5*). Robust health information systems and digital health tools provide reliable data to clinical and public health decision makers and can decrease the time from disease detection to response at the patient and national levels (*6*,*7*). Integrated, standards-based health information systems can add value to national public health emergency response by reducing redundant efforts, thus increasing efficiency, which is especially useful in resource-constrained settings (*8*,*9*).

As the COVID-19 pandemic progressed in many PEPFAR-supported countries, the PEPFAR Technical Guidance in Context of COVID-19 Pandemic publications provided strategic direction for leveraging PEPFAR investments for the pandemic response (*10*,*11*). PEPFAR-funded PCR platforms for HIV viral load testing, and related laboratory information systems, were used for SARS-CoV-2 confirmatory testing (*12*,*13*). HIV and SARS-CoV-2 testing integration occurred on both centralized high-throughput PCR instruments and decentralized point-of-care and near–point-of-care devices (*14*).

When the COVID-19 pandemic emerged, several PEPFAR-supported countries assessed the surveillance data and visualization needs of the national response (*14*,*15*). These countries rapidly assessed existing PEPFAR-funded HIV/AIDS, TB, and national health information systems and evaluated how these extensive systems could support COVID-19 surveillance data collection, analysis, visualization, and reporting needs. PEPFAR stakeholders recognized that existing standards-based, PEPFAR-funded components of their national health information systems could be enhanced to provide timely, high-quality data for national COVID-19 public health decision makers.

We describe how investments to establish and maintain standards-based health information systems for HIV/AIDS and TB in resource-constrained settings can have broader effects on the health system. Beyond their original scope, these systems can be leveraged to meet data needs for additional or emerging public health threats (*2*). We describe the methods and findings of rapid landscape assessments conducted by project teams at the US Centers for Disease Control and Prevention (CDC) headquarters in Atlanta, Georgia, USA and the CDC country office in Haiti, a PEPFAR-supported country with a long history of health information system investments. In addition, we describe results from the implementation, enhancement, and use of existing PEPFAR-supported national health information systems, electronic medical records (EMRs), and laboratory information systems for surveillance in support of the COVID-19 pandemic response in Haiti. We also discuss the centrally developed health information systems solutions designed and developed at CDC to potentially support COVID-19 surveillance requirements in select PEPFAR countries.

## Methods

### CDC, CDC Haiti, and PEPFAR Overview

**Figure 1:**
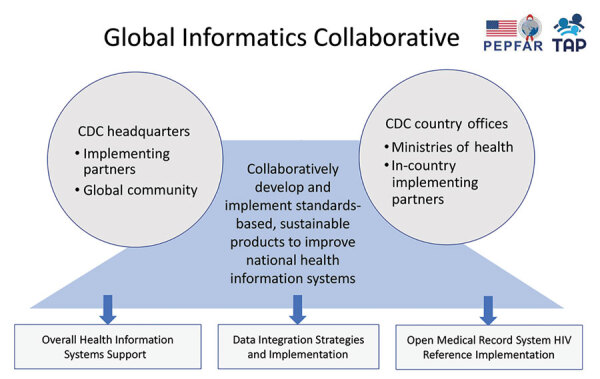
Elements of the Global Informatics Collaborative of PEPFAR-supported systems leveraged for COVID-19 pandemic response. The US CDC headquarters project team coordinated work across 3 implementing partners. Partners enhanced and customized existing PEPFAR health information systems by leveraging ongoing efforts of the TAP, a central mechanism that enables PEPFAR and national health information systems stakeholders to come together as the Global Informatics Collaborative. CDC, Centers for Disease Control and Prevention; PEPFAR, US President’s Emergency Plan for AIDS Relief; TAP, Technical Assistance Platform.

At CDC, we coordinated efforts with CDC country offices and worked with respective ministries of health in some PEPFAR countries to enhance HIV/AIDS and TB health information systems and the policies, capacities, and relationships to support COVID-19 surveillance (*15*). Our strategy was to leverage existing PEPFAR and national digital health investments to support needs beyond the initially funded diseases. Over the years, PEPFAR investments have helped countries develop a health information exchange, national data repository, and patient identity management systems. Additional central investments include an open-source EMR system, called Open Medical Record System (OpenMRS, https://wiki.openmrs.org) and the OpenMRS HIV Reference Implementation (OHRI) package to specifically support HIV/AIDS electronic medical record keeping and reporting. Technical enhancement and customization of existing PEPFAR health information systems were coordinated and funded by CDC by leveraging ongoing efforts of the Technical Assistance Platform (TAP). TAP is a central mechanism that enables PEPFAR and national health information system stakeholders to come together as the Global Informatics Collaborative (GIC) (Figure 1). 

**Figure 2:**
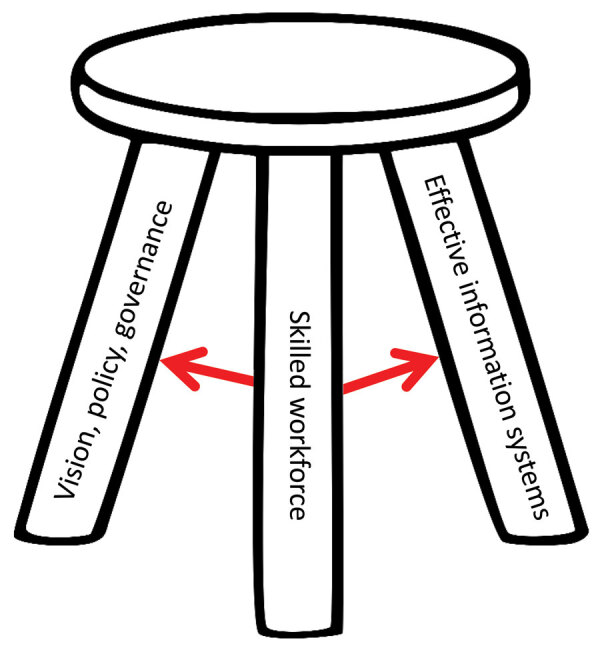
Core pillars of the US President’s Emergency Plan for AIDS Relief–supported informatics-savvy health organizations leveraged for COVID-19 pandemic response. The 3 pillars of an information-savvy health organization are supported by Technical Assistance Platform technical areas.

Building informatics-savvy health organizations is critical for tracking PEPFAR’s epidemic control goals. Information-savvy health organizations can obtain, effectively use, and securely exchange information electronically to improve public health practice and population health outcomes (*16*). Informatics-savvy health organizations have 3 core capabilities: an organization-wide informatics vision, policy, and governance; a skilled workforce; and effective information systems (Figure 2). GIC partners strategically collaborate to develop sustainable information system solutions and interventions that enable the CDC-based team to guide and assist country efforts. TAP technical areas support development of informatics-savvy health organizations in each of its 3 pillars: TAP policies and health information system governance support the vision, policy, and governance pillar; TAP workforce capacity efforts support the skilled workforce pillar; and TAP data integration strategies and implementation and OHRI support the effective information systems pillar.

**Figure 3:**
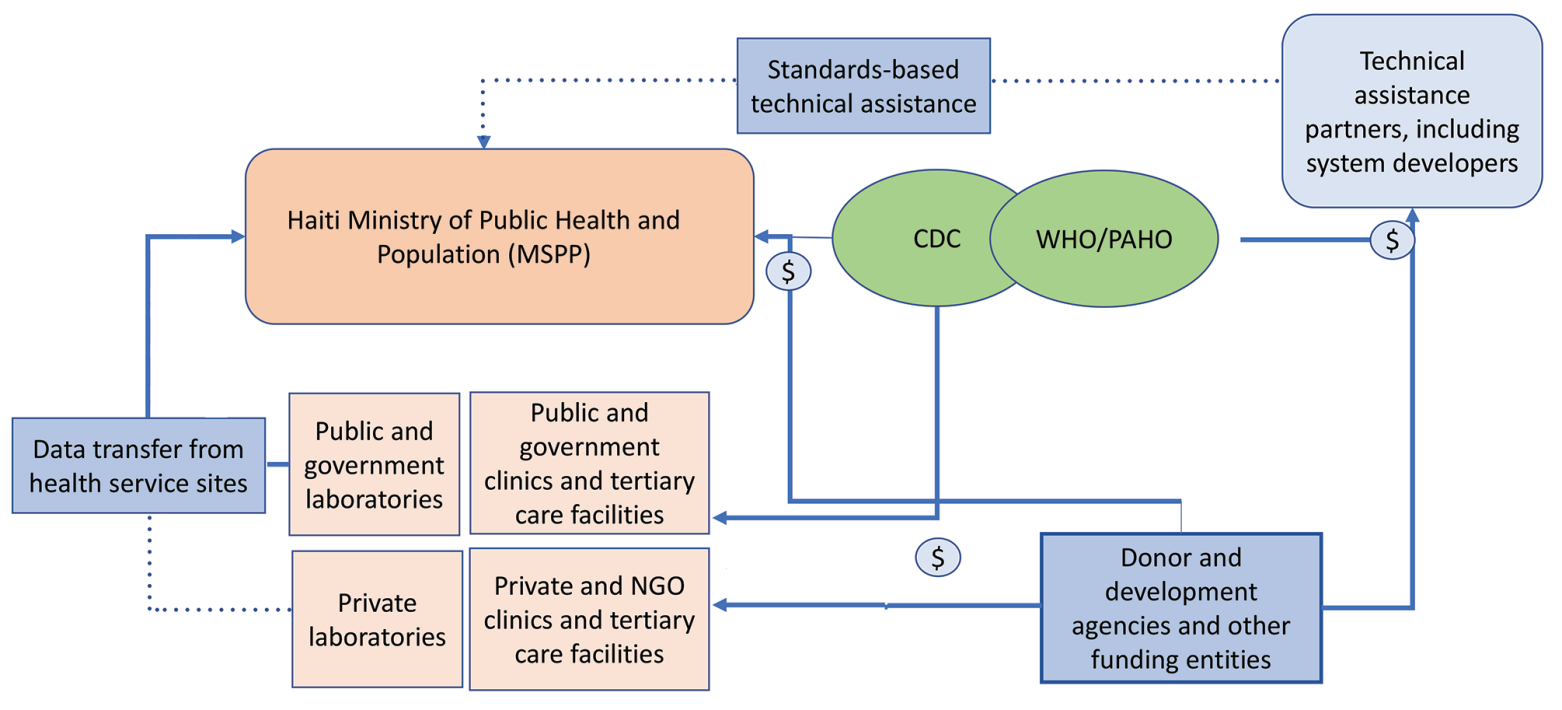
Collaborating stakeholders and beneficiaries of US President’s Emergency Plan for AIDS Relief–supported health information systems leveraged for COVID-19 pandemic response, Haiti. Funding supported Haiti’s ministry of public health and population, Ministère de la Santé Publique et de la Population (MSPP). Dollar signs denote health information systems–specific investments. Dotted lines indicate episodic or sporadic technical assistance and other inputs into the MSPP. Solid lines indicate structured technical assistance and other inputs into the MSPP’s systems. CDC, Centers for Disease Control and Prevention; NGO, nongovernmental organization; PAHO, Pan-American Health Organization; WHO, World Health Organization.

CDC staff in Haiti have been working with the country’s ministry of public health and population, Ministère de la Santé Publique et de la Population (MSPP), to strengthen public health systems by focusing on laboratory, workforce development, and health information systems (Figure 3). These cross-cutting domains are supported by leveraging several ongoing disease elimination and eradication initiatives, including initiatives for HIV/AIDS prevention and treatment, TB control, malaria elimination, lymphatic filariasis elimination, and cholera elimination. Haiti used this integrated approach and PEPFAR seed investments to establish a sophisticated HIV/AIDS health information system suite that has a central data repository that can be customized for other disease surveillance and emergency response efforts. We summarize CDC Atlanta and CDC Haiti country office experiences by outlining methods and findings from rapid assessments of existing PEPFAR and national COVID-19 surveillance health information systems.

### CDC Atlanta 

In 2021, the COVID-19 project team conducted rapid desktop landscape assessments of health information systems in Haiti and 4 PEPFAR countries in Africa by using online resources and knowledge of the countries’ health information systems through past and ongoing work. In addition, we examined activities around the 3 core TAP technical areas: data integration strategies and implementation to study surveillance data exchange; OHRI for EMR and laboratory information systems implementations and requirements; and overall health information system support to review policies, governance, and workforce capacity.

#### Summary Assessment Findings for PEPFAR Countries

All 5 study countries have implemented EMRs for HIV clinical case management. In addition, all 5 countries have laboratory information systems for HIV laboratory data management; HIV dashboards for reporting; and some form of centralized data storage at the national level for a subset of health data (e.g., HIV, COVID-19, or other reportable diseases) for supporting clinical care and public health surveillance data exchange (*15*). PEPFAR countries also deployed various digital surveillance solutions as part of the COVID-19 response, such as COVID-19 surveillance data entry systems and dashboards. In addition, most PEPFAR countries were using PEPFAR-funded laboratory infrastructure for COVID-19 testing of HIV patients and the general population.

We learned that PEPFAR-supported EMRs were not widely used for COVID-19 surveillance of HIV patients in the study countries. COVID-19 outpatient and inpatient care were usually provided at government-designated care units or private healthcare facilities that do not share health records with HIV care facilities. The failure to longitudinally share medical records is multifactorial. COVID-19 EMRs, where available, were usually standalone systems that lacked the ability to interact with national interoperability platforms to enable data exchange in support of clinical decision making (*14*). Therefore, clinicians at most COVID-19 care units did not have access to HIV-related patient risk factor information. In addition, direct exchange of COVID-19 laboratory test requests and results between EMR and laboratory information systems at a facility or through a national data repository was challenging because of gaps in system linkage and health information exchange capabilities.

Countries are exploring ways to mainstream COVID-19 clinical care (*17*), including COVID-19 care of HIV patients at PEPFAR clinics. Mainstream or longitudinal care could enable use of PEPFAR EMRs for COVID-19 outpatient assessment, surveillance, and management, including vaccination, as well as monitoring the COVID-19 burden among HIV patients. In addition, existing PEPFAR health information systems, specifically OpenMRS, laboratory information system, and country leadership support for standards-based health information exchanges provide the opportunity for leveraging PEPFAR investments to support COVID-19 surveillance (*14*). We shared assessment findings with PEPFAR countries and discussed priorities to define specific projects to address each country’s needs.

### CDC Haiti

To support COVID-19 surveillance, MSPP reviewed Haiti’s existing information systems. Haiti uses 2 central data repositories for infectious disease reporting: the national monitoring and evaluation platform, Monitoring, Évaluation et Surveillance Intégré (MESI), a national monitoring system that serves as the data hub for HIV case-based surveillance information systems; and Systeme d’Information Sanitaire Unique, a DHIS2-based (https://dhis2.org) hub for aggregate case reporting by disease and geography.

#### MESI Platform Applications and Data Flow 

The MESI platform serves as a central repository for patient records coming from facilities that use iSante/iSantePlus (OpenMRS-based EMR), which is used by >90% of health facilities supported by PEPFAR. The other 10% of health facilities use a customized in-house EMR and an EMR built on OpenMRS, from which data are transformed and uploaded into the MESI platform for data merging and removal of duplicate information. EMR data are pushed to the MESI central repository by using a network secure file transfer protocol. The data are then concatenated and cleaned, patient data merged, and duplicate data removed for a single record per person within the final dataset.

**Figure 4:**
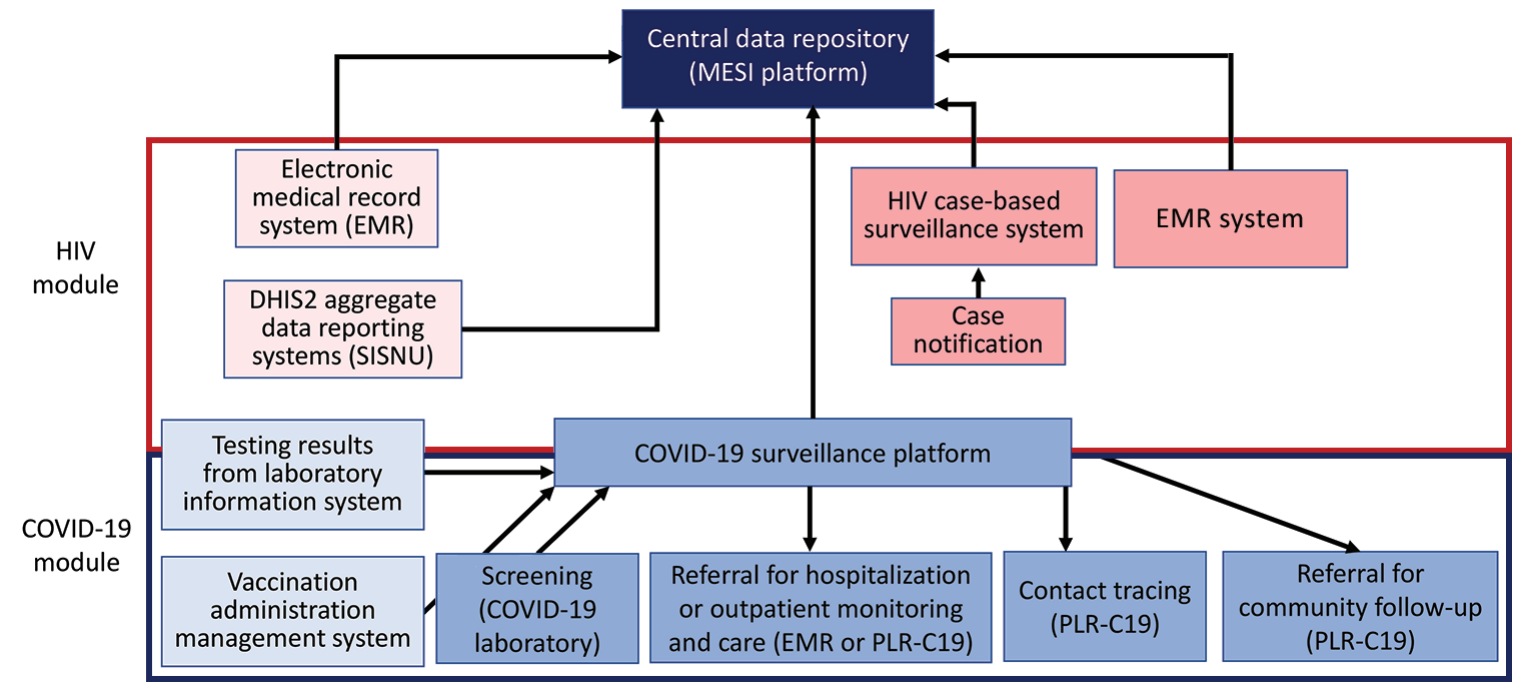
US President’s Emergency Plan for AIDS Relief–supported health information suite leveraged for COVID-19 pandemic response, Haiti. The system was built on the nation’s existing monitoring and evaluation platform, MESI. Red indicates existing HIV systems; blue indicates COVID-19 systems. SISNU is a DHIS2 (https://dhis2.org) hub for aggregate case reporting by disease and geography. C19, COVID-19; EMR, electronic medical record; MESI, Monitoring, Évaluation et Surveillance Intégreé; PEPFAR, US President’s Emergency Plan for AIDS Relief; PLR, Patient Locator and Retention mobile phone application; SISNU, Systeme d’Information Sanitaire Unique.

MESI interfaces with 3 community-level applications, generating additional patient-level data accessible on smartphones, tablets, and desktop devices. One application for tracking and tracing HIV patients and their contacts was leveraged for COVID-19 contact tracing during the pandemic response. Community health workers, Field Epidemiology Training Program graduates and residents, and some health facility managerial staff use a mobile application to routinely upload community data into the MESI platform. The mobile application has geolocation for locating persons and relational functionalities to link cases to their exposed contacts, which were critical components of the COVID-19 systems model in Haiti (Figure 4).

## Implementations and Results

### CDC Atlanta 

**Figure 5:**
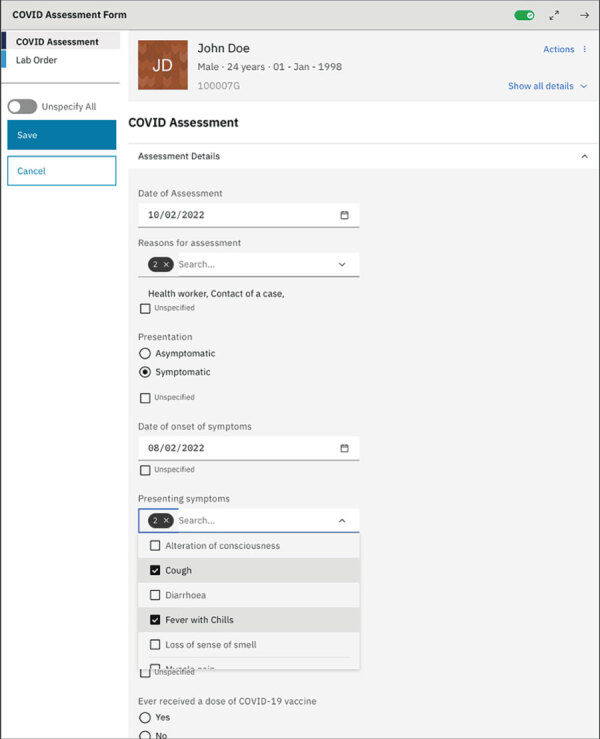
Example of COVID-19 pandemic response patient management and surveillance package developed from US President’s Emergency Plan for AIDS Relief (PEPFAR)–supported systems leveraged for COVID-19 pandemic response. The open medical record system HIV reference implementation (OHRI) platform is based on requirements from some PEPFAR countries. The COVID-19 system developed at the US Centers for Disease Control and Prevention leveraged the OHRI platform, already developed and being adapted by some PEPFAR countries.

The CDC Atlanta team studied assessment findings to identify generic national COVID-19 surveillance needs and develop requirements for a new CDC-based generic TAP product or enhancements for existing products. The team identified technical developments that could enhance PEPFAR health information systems to support COVID-19 surveillance data capture and exchange between EMR and laboratory information systems and to visualize clinical and laboratory data (*15*). The project team developed plans for enhancements by leveraging health information system strengths identified during these assessments. System enhancements were made to existing clinical and laboratory dataflows, including COVID-19 clinical data capture and laboratory request form submission within the EMR, transmission of laboratory results to EMR, and surveillance case reporting from the EMR. We developed the architecture and data entry forms for the COVID-19 package within OHRI based on OpenMRS 3.0 framework (Figure 5) and deployed it in a technical demonstration environment. The architecture used unique patient identifiers or client registries to assist with health information exchange. After ongoing testing, the open-source products were available to the GIC for country-specific customization and in-country implementation through CDC support to local resources.

### CDC Haiti

Haiti used PEPFAR-funded HIV systems for healthcare facilities and for community-based COVID-19 case management and selected their DHIS2-based system for the COVID-19 vaccine registry. Haiti leveraged an existing interoperability solution for data sharing via a health information exchange across the 2 hubs, ensuring capacity for seamless and timely COVID-19 reporting.

**Figure 6:**
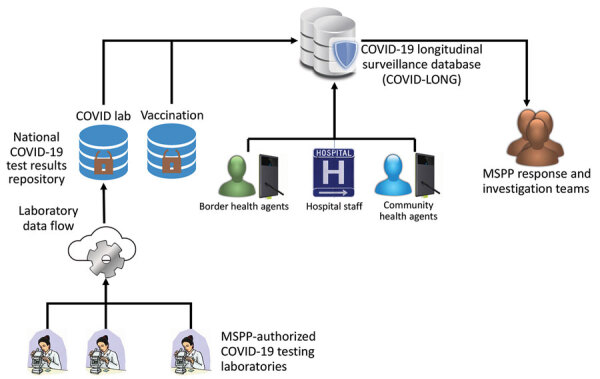
Dataflow of COVID-LONG system developed from US President’s Emergency Plan for AIDS Relief–supported HIV surveillance systems and used for COVID-19 pandemic response, Haiti. Testing laboratories, vaccination sites, hospitals, community, and border health facility agents uploaded data to the web-based system that was accessible by MSPP staff. COVID Lab, national dashboard of COVID-19 laboratory testing in Haiti; COVID-LONG, COVID-19 longitudinal surveillance database; MSPP, Ministère de la Santé Publique et de la Population.

Using US Coronavirus Aid, Relief, and Economic Security (CARES) Act (P.L. 116–136) funding to enhance existing systems and develop new information systems, some PEPFAR systems were replicated for COVID-19 surveillance, and a laboratory component was added. Previously collected paper-based COVID-19 data were retrospectively entered into the system, and subsequent newly identified cases and their contacts were entered in real time. The system included a dashboard with process and outcome indicators (Table 1). The system enabled custom analyses and data disaggregation by demographic and clinical variables and grouped results by index case for all reported and entered contacts (Figure 6).

**Table 1:** COVID-19 surveillance indicators leveraged from PEPFAR-supported systems during the COVID-19 pandemic response

Key indicators
No. persons screened
No. persons screened but not tested
No. tests without results
No. confirmed cases
No. confirmed cases hospitalized
No. confirmed cases followed at home
No. confirmed cases who recovered
No. confirmed cases who died
Time interval between confirmation and linkage to care

*PEPFAR, United States President’s Emergency Plan for AIDS Relief.

#### COVID-19 Testing System 

By September 30, 2021, the COVID-19 testing system contained 216,015 entries and 15 variables across 31 MSPP-approved testing sites. The system reported 14,711 positive test results, representing 65% of cumulatively reported cases. These data reflect a policy gap in mandatory laboratory reporting for class one notifiable diseases, especially novel etiologic agents.

COVID-19 Clinical Surveillance System 

The COVID-19 surveillance system contained 22,431 positive cases, representing 94% of cumulatively reported cases. This surveillance database also contained 375 recorded deaths among persons with a positive COVID-19 test result, and 209 reported deaths among persons who did not have a documented COVID-19 test or result. The total deaths recorded in the COVID-19 surveillance database represented 85% of cumulative reported COVID-19 deaths in the country. Forty deaths were reported among cases with a negative test result, and 19 of these persons were reported contacts of an index case. The system reported 594 exposure contacts from 407 confirmed index cases and an additional 156 reported exposure contacts from persons with negative or missing test results.

Utility of the COVID-19 Information Systems for Response Monitoring 

**Figure 7:**
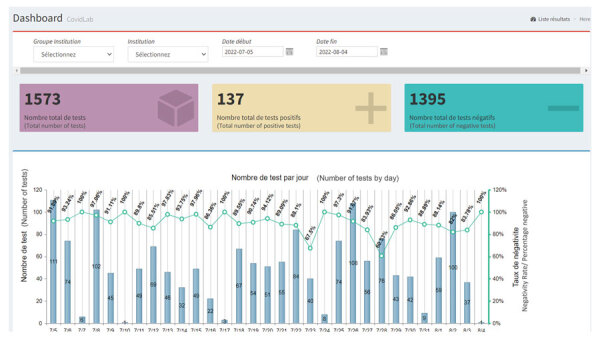
Surveillance dashboard database built from US President’s Emergency Plan for AIDS Relief–supported systems leveraged for COVID-19 pandemic response, Haiti. A screenshot from the COVID-19 interactive dashboard from Haiti COVID-19 surveillance database shows a COVID-19 histogram tracking the number of COVID-19–positive cases per day on the top row and positivity rates of total reported COVID-19 tests on the bottom row.

Despite challenges with data completeness and reporting gaps (largely from the private laboratory network), the COVID-19 health information system provided critical data for national COVID-19 decision-making. The dashboard showed the number of positive cases per day and positivity rates of total reported COVID-19 tests (Figure 7). This dashboard was built by leveraging the HIV dashboard used to track patient retention in HIV care. On the basis of positivity rates, the dashboard data assisted staff and decision makers with supply management for COVID-19 testing commodities and allocation of therapeutic treatment and human resources. When the data were disaggregated by department, staff and decision makers were able to allocate resources by geographic area. As the dashboard’s effectiveness became evident, we observed a 31% increase in system use over 90 days.

## Discussion

The COVID-19 pandemic response required rapid availability of surveillance data, which necessitated multisectoral response efforts and internal and external stakeholder participation. Setting up a new health information system for any disease takes considerable effort and time and involves high-level strengths and needs assessments, requirement development, resource allocation, technical development, pilot testing, training, and implementation. We describe efforts in a PEPFAR-supported country and concurrent CDC Atlanta work for technical enhancements of existing standards-based PEPFAR health information systems after rapid landscape assessments of system strengths and needs. Our approach was consistent with principles for digital development (*18*), including rapid and cost-effective implementation; data standardization, integration, and reporting; and local sustainability and community support (*18*).

PEPFAR and national investments enabled some countries to allocate resources to expand or enhance existing health information systems to rapidly support the national COVID-19 response, which improved the timeliness and usefulness of data for decision making. PEPFAR health information system enhancements and new products supported COVID-19 clinical case management, surveillance, laboratory results, and dashboards (Table 2).

**Table 2:** COVID-19 response support leveraged from PEPFAR investments

Program area	CDC headquarters	CDC Haiti office
Clinical case management	OHRI enhanced by developing a COVID-19 module for case management and surveillance at healthcare facilities	PEPFAR-funded HIV systems were used for healthcare facilities and community-based COVID-19 case management.
Surveillance	Enhanced national health information exchange model was used to link electronic systems for COVID-19 case confirmation and case management	Existing interoperability solutions were leveraged for data sharing via a health information exchange across 2 national COVID-19 data hubs
Laboratory	Automated exchange functionality was developed for COVID-19 testing requests and results between EMR and local laboratory information systems directly at a facility or through a national data repository	PEPFAR systems were replicated for COVID-19 surveillance, and a laboratory component was added for COVID-19 laboratory data flow
Dashboard	Dashboard requirements were developed for specific indicators, such as the number of persons living with HIV who were hospitalized for COVID-19	COVID-19 surveillance dashboard was built by leveraging the HIV dashboard used to track patient retention in HIV care

*CDC, US Centers for Disease Control and Prevention; EMR, electronic medical record; OHRI, Open medical record system HIV reference implementation; PEPFAR, United States President’s Emergency Plan for AIDS Relief.

Enhanced reporting reduced time needed to make data available. Timeliness of data improved decision making capacity for resource allocation, identification of hot spots, and other transmission factors for mitigation measures. Enhanced reporting also enabled surveillance for new variants and other factors affecting virus transmission. In addition, enhanced reporting enabled validation of novel diagnostic tools, instruments, and treatment efficacy and monitoring of response outcomes at the system and patient levels.

The COVID-19 project team at CDC Atlanta incorporated COVID-19 surveillance requirements into the existing TAP product planning to develop the OHRI–COVID-19 module and health information exchange architecture design. The team developed this module to enable integrated COVID-19 surveillance for HIV patients in PEPFAR countries. As countries move toward mainstream COVID-19 care, the team has been testing various implementation use cases. One use case would enable COVID-19 surveillance for HIV patients by implementing OHRI–COVID-19 module in healthcare facilities where PEPFAR EMRs currently are used only for HIV patients. A second use case would conduct COVID-19 surveillance for all patients by implementing the OHRI–COVID-19 module in healthcare facilities where PEPFAR EMRs are being used for all patients.

Despite challenges with implementing mandated reporting, by showing the usefulness of the testing and surveillance databases, CDC Haiti secured support from the MSPP minister and the broader government of Haiti via the President’s Commission on SARS-CoV-2 Co-Chairs. As Haiti’s sole government-mandated health authority, MSPP has responsibility for implementing and ensuring internationally acceptable standards for health data and the health information systems through which the data are collected, stored, managed, accessed, and used (*19*). Leveraging PEPFAR-funded flexible, adaptable, and customizable health information systems enabled MSPP to build on centrally warehoused data infrastructure for the COVID-19 response, in keeping with internationally acceptable standards. Although the experience provided evidence for health policy reform, particularly for peripheral systems out of compliance, emerging challenges with timeliness and completeness of data entry compromised the usefulness of warehoused SARS-CoV-2 data at the system’s initiation. In addition, challenges during the transition from paper-based forms to electronic data entry created a lag in cumulative reporting.

We learned that several factors enabled success in CDC Haiti and CDC Atlanta work. Haiti had high level decision-makers actively engaged in the project and the local health ministry served as a de facto International Standards Organization, which reinforced CDC Haiti and World Health Organization defined standards for data systems. The health ministry’s lack of official International Standards Organization status did impede its ability to ensure comparable standards for privately owned and implemented health information systems. MSPP already had standards-based health information systems in place and had technically skilled staff to use the systems and implement changes.

In conclusion, accurate and timely COVID-19 surveillance data were needed to understand COVID-19 epidemiology for HIV patients and determine how to manage the pandemic, based on models similar to those used for HIV (*5*). CDC’s efforts to enhance PEPFAR-supported information systems during the COVID-19 pandemic included expanding HIV and TB EMRs for COVID-19 case management, vaccination, surveillance, and case reporting; enhancing surveillance through reporting of laboratory test results; strengthening national data repository to facilitate data exchange for enhanced surveillance; and improving dashboards for decision makers. The use and enhancement of existing PEPFAR health information systems for COVID-19 response showed that investing in establishing and maintaining health information systems in resource-constrained settings can positively impact health systems beyond the original scope (Table 3).

**Table 3:** Enabling factors of informatics-savvy health organizations leveraged by CDC headquarters and CDC country offices for COVID-19 pandemic response*

Pillars and supporting functions
Pillar 1. Vision, policy, and governance
Acceptance by country leadership
Ownership by host country governments
Timely stakeholder engagement to maximize uptake and utility
Collaboration among implementing partners and alignment of various stakeholders’ priorities, activities, and plans
Use of existing standards-based data systems for routine health service delivery and surveillance
Assured confidentiality and trust for new, name-based data systems, specifically for novel infections and other highly stigmatized conditions
Central coordination of health information system investments
Pillar 2. Skilled workforce
Local capacity building for systems development
Use of existing investments in easily customizable health information systems solutions built on open-source platforms ensured the availability of local technical capacity
Availability of strong technical capabilities within the country
Pillar 3. Effective information systems
Investments in interoperability solutions to facilitate health information exchange and integrate data across systems and disease programs
Existing investments in flexible and scalable IT infrastructure
Use of existing standards-based open-source electronic medical record platforms

*CDC, US Centers for Disease Control and Prevention.
